# Decreasing brown bear (*Ursus arctos*) habitat due to climate change in Central Asia and the Asian Highlands

**DOI:** 10.1002/ece3.4645

**Published:** 2018-11-20

**Authors:** Junhu Su, Achyut Aryal, Ibrahim M. Hegab, Uttam Babu Shrestha, Sean C. P. Coogan, Sambandam Sathyakumar, Munkhnast Dalannast, Zhigang Dou, Yila Suo, Xilite Dabu, Hongyan Fu, Liji Wu, Weihong Ji

**Affiliations:** ^1^ College of Grassland Science, Key Laboratory of Grassland Ecosystem (Ministry of Education) Gansu Agricultural University Lanzhou China; ^2^ Gansu Agricultural University–Massey University Research Centre for Grassland Biodiversity Gansu Agricultural University Lanzhou China; ^3^ Institute of Natural and Mathematical Sciences Massey University Auckland New Zealand; ^4^ Department of Hygiene, Zoonoses and Animal Behaviour & Management, Faculty of Veterinary Medicine Suez Canal University Ismailia Egypt; ^5^ Institute for Agriculture and the Environment University of Southern Queensland Toowoomba QLD Australia; ^6^ The Charles Perkins Centre, School of Biological Sciences The University of Sydney Sydney Australia; ^7^ The Department of Renewable Resources University of Alberta Edmonton AB Canada; ^8^ Wildlife Institute of India Chandrabani India; ^9^ Bats Research Center of Mongolia Ulaanbaatar Mongolia; ^10^ Gansu Yanchiwan National Nature Reserve Bureau Subei China

**Keywords:** Asian highlands, brown bear, Central Asia, climate change, habitat shift, species distribution model

## Abstract

Around the world, climate change has impacted many species. In this study, we used bioclimatic variables and biophysical layers of Central Asia and the Asian Highlands combined with presence data of brown bear (*Ursus arctos*) to understand their current distribution and predict their future distribution under the current rate of climate change. Our bioclimatic model showed that the current suitable habitat of brown bear encompasses 3,430,493 km^2^ in the study area, the majority of which (>65%) located in China. Our analyses demonstrated that suitable habitat will be reduced by 11% (378,861.30 km^2^) across Central Asia and the Asian Highlands by 2,050 due to climate change, predominantly (>90%) due to the changes in temperature and precipitation. The spatially averaged mean annual temperature of brown bear habitat is currently −1.2°C and predicted to increase to 1.6°C by 2,050. Mean annual precipitation in brown bear habitats is predicted to increase by 13% (from 406 to 459 mm) by 2,050. Such changes in two critical climatic variables may significantly affect the brown bear distribution, ethological repertoires, and physiological processes, which may increase their risk of extirpation in some areas. Approximately 32% (1,124,330 km^2^) of the total suitable habitat falls within protected areas, which was predicted to reduce to 1,103,912 km^2^ (1.8% loss) by 2,050. Future loss of suitable habitats inside the protected areas may force brown bears to move outside the protected areas thereby increasing their risk of mortality. Therefore, more protected areas should be established in the suitable brown bear habitats in future to sustain populations in this region. Furthermore, development of corridors is needed to connect habitats between protected areas of different countries in Central Asia. Such practices will facilitate climate migration and connectivity among populations and movement between and within countries.

## 1 INTRODUCTION

Around the world, climate change has had significant direct and indirect impacts on terrestrial species, by being a major cause of speciation and species extirpation (Pound & Salzmann, 2017). Many studies have recently focused on the ecological (Etterson & Mazer, 2016; Wikelski & Tertitski, 2016), ethological (Munoz, Marquez, & Real, 2015) and biological changes (Torres‐Diaz et al., 2016; Hulme, 2016) in relation to climatic change. For example, various ecosystems are vulnerable to climate change which may induce a broad array of adverse effects such as disturbances of phenological events, food web disruptions, pathogens and disease spread and ultimately, in worst case scenarios, may include extinction risks (Wu, Lu, Zhou, Chen, & Xu, 2016). Furthermore, climate change has impacted species distributions by reducing and fragmenting of the area of animal habitats (Chen, Hill, Ohlemuller, Roy, & Thomas, 2011; Loarie et al., 2008; Lord & Whitlatch, 2015; Lundhede et al., 2014; Su, Aryal, Nan, & Ji, 2015; Wu, 2016). However, to tackle the challenge of the changing climatic conditions, species have adopted different mechanisms to counteract the magnitude and speed of climate change either individually or within a population (Hill, Griffiths, & Thomas, 2011). For example, natural populations may react to climate change either collectively by shifting their geographical habitats (Hoffmann & Sgro, 2011), or individually by adjusting their behavioral activities through modifications of their diet, activity and energy budget and reproductive tactics (Bellard, Bertelsmeier, Leadley, Thuiller, & Courchamp, 2012). Although these tactical responses have proven to have a short‐term efficiency (Crane, Roncoli, & Hoogenboom, 2011) to withstand climatic changes, some studies showed that up to 42% of species in certain geographical areas are at risk of extinction in the long term due to deforestation and habitat fragmentation solely (Sodhi, Koh, Brook, & Ng, 2004).

The Asian highlands, the high mountainous areas of Afghanistan, Bhutan, China, India, Mongolia, Nepal, Pakistan and Russia, contain rich biological diversity and provide important ecosystem services for downstream human communities. The region has also some of the greatest species endemism on the planet and the great variation in climate, topography, and elevation underpins rich cultural diversity (Xu et al., 2009). However, climate change has greatly impacted both biological diversity and ecosystems services in these areas (Aryal, Brunton, & Raubenheimer, 2014; Kujala, Moilanen, Araujo, & Cabeza, 2013; Xu et al., 2009). The variation of climate effects that have been detected in the Asian Highlands shows progressive substantial changes at several levels from species to ecosystems (Yu, Luedeling, & Xu, 2010). Impacts of climate change on the hydrology, biodiversity, and ecosystems in this area have been reported which include glacial melting, changes in streamflow, groundwater scarcity, altitudinal shifts, reduction in plant and animal habitats, biodiversity loss, and grassland desiccation (Pressey, Cabeza, Watts, Cowling, & Wilson, 2007; Shrestha & Bawa, 2014; Walther et al., 2002). Furthermore, many studies foresee that future climate change would have even greater impacts on biodiversity in Central Asia (Chen, Li, Deng, Fang, & Li, 2016; Garcia, Cabeza, Rahbek, & Araujo, 2014; Zhang, Zhang, & Sanderson, 2013). It is important to understand the results of these changes in terms of habitat composition, structure and function and the responses of animal geographical distribution, which can guide conservation actions and government efforts in the Asian Highlands in response to these changes (Xu & Grumbine, 2014).

**Figure 1:**
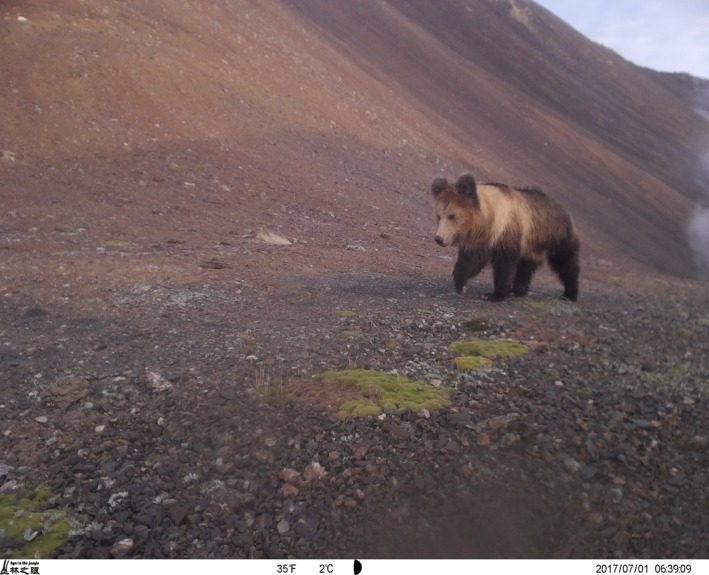
Brown bear (*Ursus arctos*)

Brown bear (*Ursus arctos*) is a solitary, non‐territorial species with a promiscuous or polygamous mating system (Jerina, Jonozovic, Krofel, & Skrbinsek, 2013; Figure 1). It has a circumglobal distribution in the northern hemisphere, occurring in North America (The United States and Canada), Europe, and northern and Central Asia (McLellan et al., 2008). Many of these regions are experiencing rapid climate change (Shrestha, Gautam, & Bawa, 2012). In Central Asia and the Asian Highlands, brown bear distribution is mostly limited to higher elevation areas where more pronounced effects of climate change have been reported (Aryal, 2012; Aryal et al., 2014). In these regions, brown bear distribution and presence may be impacted by changing thermal regimes, vegetation, and prey abundance. Such changes may potentially increase human–bear conflicts due to shifting in distribution of resources and possibly increasing scarcity of key resources such as water and food. In the same way, decrease in species diversity in the region due to climate change (Bertelsmeier, Luque, & Courchamp, 2013; Seim et al., 2016) might directly affect the distribution and abundance of the brown bear by reducing food resources. Reduced food abundance may result in increased incidences of brown bears moving to anthropogenic areas in search of food, which could lead to increased levels of livestock depredation and human–bear conflict. However, little is known regarding how brown bears habitats will be affected by future climate change in this region. To date, there are few published studies which assess the potential impacts of climate change on brown bears and their habitats (Roberts, Nielsen, & Stenhouse, 2014), and none in Asia. However, in recent years, a growing interest in the ecological and evolutionary mechanisms of habitat change due to climate change has promoted development of new models for predicting biodiversity futures (Anderson, 2013). Here, we model the current suitable habitats for brown bears and predict the change in their future distribution due to climate change in Central Asia and the Asian Highlands. We used bioclimatic variables and biophysical layers of Central Asia combined with presence data of brown bear to understand their current and future distribution. Such information will be helpful in managing brown bear populations and designing future conservation policies in Asia.

## 2 MATERIAL AND METHODS

### 2.1 Study area and presence data

We selected 11 countries of Central Asia and the Asian Highlands where brown bears are reported to occur (McLellan et al., 2008). The countries included are Afghanistan, Bhutan, China, India, Kazakhstan, Kyrgyzstan, Mongolia, Nepal, Pakistan, Tajikistan, and Uzbekistan. We obtained brown bear presence data by field surveys, information from published and unpublished sources, and occurrence data from Global Biodiversity Information Facility (GBIF; https://www.gbif.org). Presence data were collected through field surveys in Nepal (2007–2011), India (2001, 2006, 2012), Mongolia (2010–2014), and China (2014). In these studies, brown bear presence was recorded by sign survey including camera traps, scats, and tracks. Brown bear presence information from published and unpublished literatures was obtained for India (Sathyakumar, 2001, 2006 ; Sathyakumar, Kaul, Ashraf, Mookherjee, & Menon, 2012), Pakistan (Nawaz, 2007; Nawaz, Swenson, & Zakaria, 2008), Mongolia (Mccarthy, Waits, & Mijiddorj, 2014), China (Gong & Harris, 2006, Xu, 2010), Nepal (Aryal, Iii, Raubenheimer, Ji, & Brunton, 2012; Aryal, Sathyakumar, & Schwartz, 2010), Kazakhstan (Loginov, 2012), and Afghanistan (Moheb, Lawson, & Mostafawi, 2012). Additionally, information on brown bear presence, recent killing/poaching sites, and locations of human–bear encounters and conflicts was obtained from local authorities.

We used a species distribution map from the International Union for Conservation of Nature (IUCN; McLellan et al 2008; IUCN & Wildlife Conservation Society, 2008), to extract brown bear distribution areas (resident) for Asia with the help of ArcGIS. We plotted GPS points of current presence data we obtained and compared with the current IUCN distribution map for ground truthing for validation and correction. We downloaded protected areas of Asia (IUCN & UNEP‐WCMC, 2014), and based on the literature survey of brown bear presence, we selected areas and overlaid them into the species distribution map. Only the brown bear occurrence points within protected areas were selected for further analysis to model the potentially suitable habitats for brown bear. We removed the unconfirmed brown bear distribution areas (potential distribution) which lay outside of protected areas throughout the range on which we did not have evidence of presence. In this way, we validated brown bear presence points collected from field surveys and from literature and used them for final analysis. We also created 500 presence points within each protected area (one point per 5 km × 5 km cell) that overlaid with the IUCN's brown bear distribution map using Hawth's tools extension in ArcGIS. We verified the randomly generated presence points by brown bear experts from China, Mongolia, Nepal, and India. We did field verification of those randomly generated points in China, and Nepal by consulting with local park authorities to determine the current brown bear presence and removed those points from the analysis where absence was indicated. We also removed duplicate presence points and retained only one presence points per 1‐km^2^ grids cells. After validation, verification of presence points and removal of duplicates, we used remaining 209 occurrence points of brown bear presence for further analysis.

### 2.2 Bioclimatic data

We used nineteen bioclimatic variables derived from www.worldclim.org (Hijmans et al., 2005), land use land cover data from global land cover data ( https://www.glcn.org; Latham et al., 2014) and altitude from GTOPO30 ( https://lta.cr.usgs.gov/GMTED2010; Danielson & Gesch, 2011). We prepared aspect and slope layers using a digital elevation model (DEM) layer using ArcGIS and clipped all variables to our study areas. We then extracted the values of each variable corresponding to the species occurrence locations to perform correlation analysis and removed highly correlated variables (>0.85; Table 1). We used the remaining 14 variables for our final analysis.

**Table 1:** Relative contribution of environment variable to the MaxEnt model

Variables	Percent contribution	Permutation importance
Annual Mean Temperature (BIO1)	43.9	58.3
Mean Temperature of Wettest Quarter (BIO8)	27.1	0.2
Precipitation of Driest Month (BIO14)	5.2	2.7
Min Temperature of Coldest Month (BIO6)	4.4	2.2
Annual Precipitation (BIO12)	4.3	1.7
Elevation	4.3	7.3
Aspect	2.3	1.7
Temperature Annual Range (BIO7)	2.1	14.4
Slope	2.1	0.8
Land cover	1.2	1
Precipitation Seasonality (BIO15)	1	4.7
Mean Diurnal Range (BIO2)	0.8	1.5
Isothermality (BIO3)	0.7	2.8
Precipitation of Coldest Quarter (BIO19)	0.5	0.7

For projections of future climate, we used the MIROC5 (Model for Interdisciplinary Research On Climate) General Circulation Model (GCM) developed by the University of Tokyo, National Institute for Environmental Studies, Japan Agency for Marine‐Earth Science and Technology (Sperber et al., 2013; Sharmila, Joseph, Sahai, Abhilash, & Chattopadhyay, 2015; Mishra et al., 2014). The GCM data were downscaled using the delta method and bias corrected by worldclim's current climate ( https://worldclim.org/). We ran the MIROC5 model using the Representative Concentration Pathway 4.5, a “middle‐of the‐road” GHG (Green House Gas) scenario. For our analysis, we used current and 2,050 (average for 2,041–2,060) time series climate change scenario ( https://worldclim.org/cmip5_30s).

### 2.3 Modeling current and future suitable habitat

We used maximum entropy (MaxEnt) species distribution modeling (SDM; Phillips Steven and Dudík, 2008) to map the current and predicted future distribution of brown bear in the study area. MaxEnt is a widely used tool for modeling species distributions using presence data of species and various environmental parameters (Kramer‐Schadt et al., 2013). There are limitations in MaxEnt modeling (Boria, Olson, Goodman, & Anderson, 2014; Radosavljevic & Anderson, 2013). We minimized these limitations by using validated presence data (from field surveys, past studies, and IUCN distributions map). Finally, we evaluated and selected the best model projection of current and future scenarios. Since our data were based on field surveys and areas with existing brown bear presence in protected areas, there may be some biases due to auto‐correlation of localities and variables (Boria et al., 2014); therefore, we validated the model using the area under the curve (AUC) of the receiver operator characteristic (ROC) curve to correct for biased samples and variables (Pearce et al., 2000; Roura‐Pascual, Brotons, Peterson, & Thuiller, 2009). We prepared suitable habitat of brown bear based on equal training sensitivity and specificity logistic threshold (Table 2) and removed the area below <0.39 of equal training sensitivity and specificity logistic threshold (Table 2).

## 3 RESULTS

After removing highly correlated variables (>*r* = 0.85), we used 14 variables for further analysis, such as Temperature Seasonality (BIO4), Max Temperature of Warmest Month (BIO5), Mean Temperature of Driest Quarter (BIO9), Mean Temperature of Warmest Quarter (BIO10), Mean Temperature of Coldest Quarter (BIO11), Precipitation of Wettest Month (BIO13), Precipitation of Wettest Quarter (BIO16), Precipitation of Warmest Quarter (BIO18), and Precipitation of Driest Quarter (BIO17; (Supporting information Table S1). Overall, annual and seasonal temperature and precipitation were the main bioclimatic factors contributing to brown bear habitat suitability, which together contributed more than 90% to the species distribution model (Table 1). Annual Mean Temperature (BIO1) contributed the most (43.9%), followed by Mean Temperature of Wettest Quarter (BIO8; 27.1%), Precipitation of Driest Month (BIO14; 5.2%), Minimum Temperature of Coldest Month (BIO11; 4.4%), and Annual Precipitation (BIO12; 4.3%) to the model. Aspect, slope, and land cover contributed <3% to our model (Table 1).

**Figure 2:**
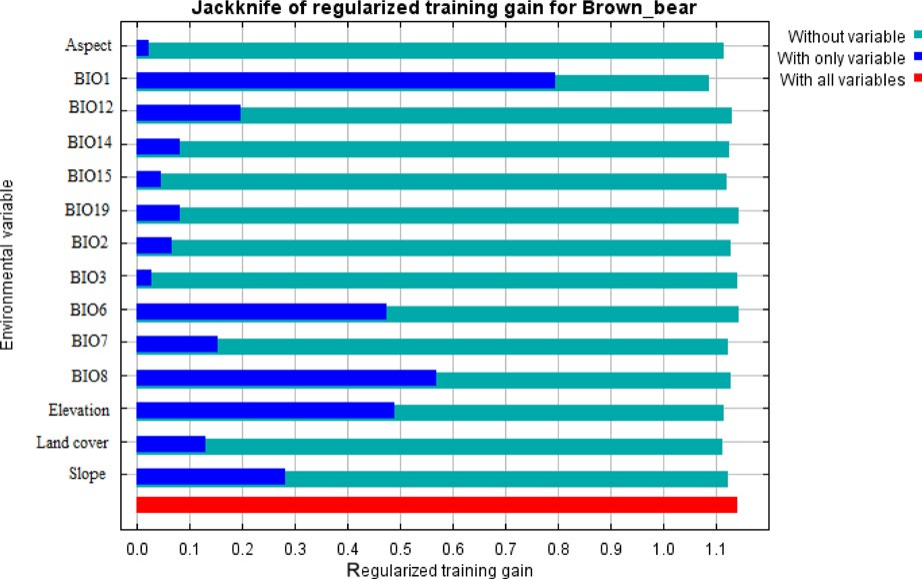
Results of the jackknife test of variable importance. The environmental variable with highest gain when used in isolation is Mean Temperature of Warmest Quarter (Bio10), which therefore appears to provide the most useful information by itself. The environmental variable that decreases the gain the most when omitted is land cover, which appears to have the most information that isn't present in the other variables

**Figure 3:**
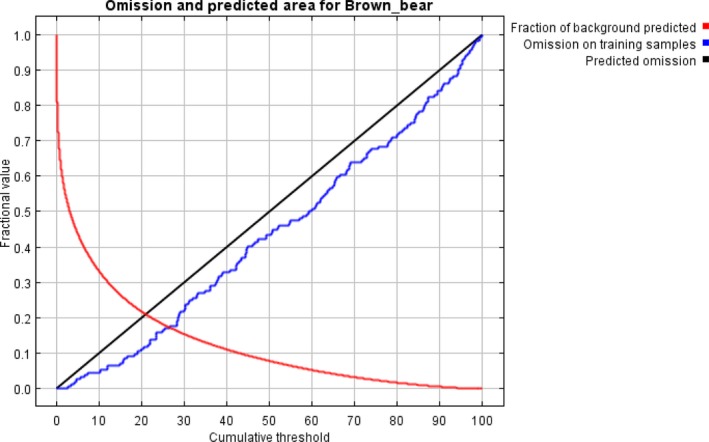
The omission rate and predicted area as a function of the cumulative threshold. The omission rate is calculated both on the training presence records, and (if test data are used) on the test records. The omission rate should be close to the predicted omission, because of the definition of the cumulative threshold

**Figure 4:**
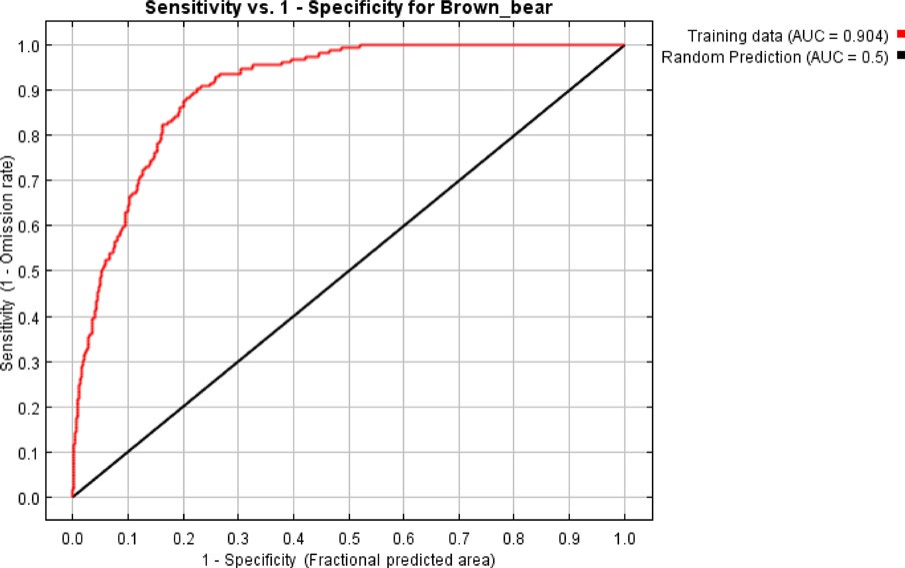
The receiver operating characteristic (ROC) curve for the same data. Note that the specificity is defined using predicted area, rather than true commission. This implies that the maximum achievable AUC is less than 1. If test data are drawn from the MaxEnt distribution itself, then the maximum possible test AUC would be 0.868 rather than 1; in practice, the test AUC may exceed this bound

**Figure 5:**
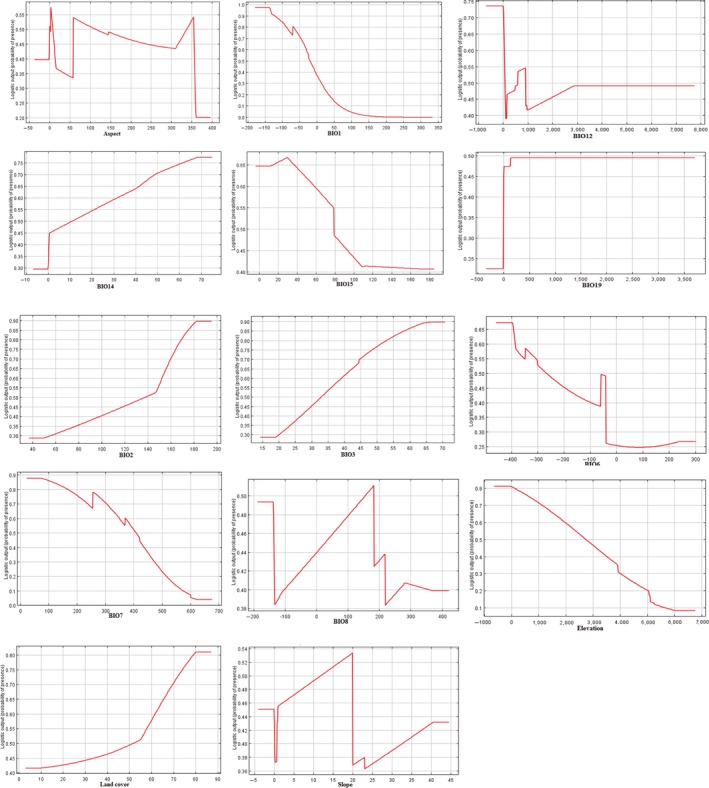
Response curves. These curves show how each environmental variable affects the MaxEnt prediction. The curves show how the logistic prediction changes as each environmental variable is varied, keeping all other environmental variables at their average sample value

The result of the jackknife test of variable importance showed that highest gain was in annual mean temperature and elevation (Figure 2). The environmental variable, which decreased the gain the most when it was omitted, was the land cover (Figure 2). Our model was well represented because the omission rate was close to the predicted omission as a function of the cumulative threshold and both were calculated based on the training presence records (Figure 3, Table 2). Our model and environmental variables described the current distribution of brown bear very well in the study area (AUC = 0.90; Figures 2, 3, 4). Response curves showed each environmental variable affected the prediction of brown bear distribution, which keeps all other environmental variables at their average sample value and showed how the logistic prediction changes as each environmental variable is varied (Figure 5).

**Table 2:** Common thresholds (cumulative and logistic) and corresponding omission rates

Cumulative threshold	Logistic threshold	Description	Fractional predicted area	Training omission rate
1.000	0.032	Fixed cumulative value 1	0.629	0.000
5.000	0.107	Fixed cumulative value 5	0.437	0.026
10.000	0.171	Fixed cumulative value 10	0.332	0.046
2.652	0.067	Minimum training presence	0.519	0.000
19.020	0.280	10 percentile training presence	0.226	0.099
26.635	0.363	Equal training sensitivity and specificity	0.173	0.171
19.871	0.292	Maximum training sensitivity plus specificity	0.218	0.105
2.652	0.067	Balance training omission, predicted area and threshold value	0.519	0.000
9.876	0.170	Equate entropy of thresholded and original distributions	0.334	0.046

Note

If test data are available, binomial probabilities are calculated exactly if the number of test samples is at most 25, otherwise using a normal approximation to the binomial. The “Balance” threshold minimizes 6 × training omission rate +0.04 × cumulative threshold +1.6 × fractional predicted area.

### 3.1 Role of temperature and precipitation on brown bear habitat

Our model showed that brown bear distribution was attributed to bioclimatic variables associated with climate change: annual temperature (BIO1) and precipitation (BIO12; Table 1). The spatially averaged current mean annual temperature of brown bear habitat was −1.2°C (maximum 17.1, minimum −13.7°C) and is predicted to increase to 1.6°C (maximum 19.9°C and minimum −11.2°C) by 2,050. Similarly, current annual mean precipitation of brown bear habitat is predicted to increase by 13%, from 406 mm to 459 mm by 2,050.

### 3.2 Suitable habitats of brown bear under current and future climates

**Figure 6:**
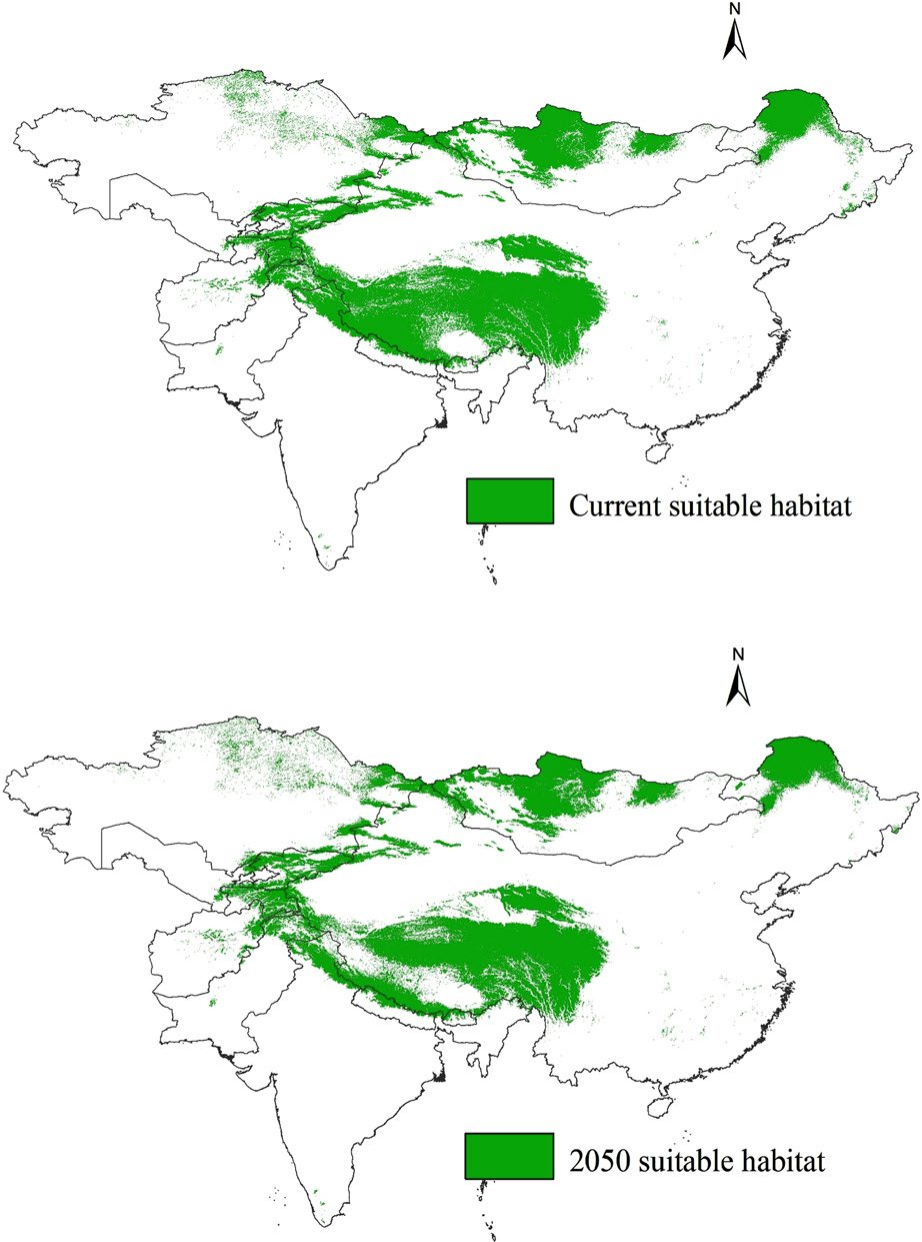
Current and future suitable habitat of brown bear in Asia

Our model showed that present suitable brown bear habitat area was 3,430,493.90 km^2^ in Central Asia (Figure 6). Most of the habitats located in China (65.7%), followed by Mongolia (13.9%), Kazakhstan (5.1%), India (4.1%), Kyrgyzstan (3.5%), and Pakistan (2.0%; Table 3, Figure 6). The least amount of suitable habitat was found in Bhutan (0.4%), Uzbekistan (0.3%), Nepal (1.2%), and Afghanistan (1.4%; Table 3, Figure 6).

**Table 3:** Current and future suitable habitat of brown bear

Country	Current suitable habitat (area in km^2^)	Current area in %	Future (2,050) suitable habitat (area in km^2^)	% of Change
Mongolia	477,503.00	13.87	465,880.00	−2.43
Afghanistan	47,474.70	1.38	42,402.30	−10.68
Kazakhstan	176,320.00	5.12	160,711.00	−8.85
Tajikistan	76,153.90	2.21	75,215.30	−1.23
Kyrgyzstan	118,768.00	3.45	111,641.00	−6.00
Uzbekistan	10,271.70	0.30	15,523.40	51.13
China	2,259,810.00	65.66	1,969,610.00	−12.84
India	141,002.00	4.10	103,882.00	−26.33
Bhutan	14,182.10	0.41	13,084.00	−7.74
Nepal	40,505.90	1.18	35,132.30	−13.27
Pakistan	68,502.60	1.99	56,501.30	−17.52
Total	3,430,493.90		3,051,632.60	−11.04

Our results showed that the current suitable habitat will be reduced by 11% (378,861 km^2^) across Central Asia by 2,050 (Table 3) due to climate change. The most suitable habitat is predicted to be lost in India (26% loss), Pakistan (17%), and Nepal (13%). In China, 290,200 km^2^ (13%) of suitable habitat is predicted to be lost by 2,050 (Table 3). However, a small increase in suitable habitat for brown bear is predicted in Uzbekistan relative to the entire study area (10,271 km^2^), but a relatively large gain within the country (51%; Table 3; Figure 6).

### 3.3 Suitable habitat within protected areas

About 1.8% of the areas from the current total suitable habitat lay within protected areas (1,124,330 km^2^), which is predicted to decrease to 1,103,912 km^2^ by 2,050 (Table 4). In some countries, however, suitable habitat loss within protected areas was greater. For example, India will experience the greatest loss at 27% of suitable habitats, followed by Tajikistan (6.8% loss). China's predicted loss within protected areas is about 12,841 km^2^ (1.4%) of a suitable area by 2,050. However, suitable habitat within protected areas is predicted to increase in some countries, such as Uzbekistan (21%) and Bhutan (9%; Table 4).

**Table 4:** Suitable habitat within protected areas current and projected for 2,050

Country	Current suitable habitat within protected area (area in km^2^)	Future (2,050) suitable habitat within protected area (area in km^2^)	% of Change
Mongolia	60,527.40	62,703.20	3.59
Afghanistan	6,290.29	5,922.30	−5.85
Kazakhstan	16,247.50	16,181.60	−0.41
Tajikistan	24,579.60	22,897.90	−6.84
Kyrgyzstan	5,990.36	5,796.69	−3.23
Uzbekistan	5,278.12	6,401.47	21.28
China	940,672.00	927,831.00	−1.37
India	33,124.80	24,162.20	−27.06
Bhutan	5,572.79	6,111.63	9.67
Nepal	18,736.80	18,597.30	−0.74
Pakistan	7,310.58	7,307.27	−0.05
Total	1,124,330.24	1,103,912.56	−1.82

## 4 DISCUSSION

Habitat use by organisms reflects the environmental characteristics that augment their fitness (Fretwell, 1969). Generally, it is supposed that a species distribution or individuals within a population is a good indicator of habitat structure and particularly manifests their preference toward the habitat qualities. Predictive modeling has become a valuable tool for successful conservation planning or wildlife management through identification and prediction of habitat appropriateness for given species (Schadt et al., 2002). Indeed, predicting future geographical range by species distribution modeling is pivotal to understand their ecological requirements and biological responses to upcoming climatic changes (Duan, Kong, Huang, Fan, & Wang, 2014).

Across Central Asia, suitable habitats of the brown bear are widely distributed in higher elevation regions and are predicted to moderately decrease by 2,050 due to climate change; however, the extent of the change is not felt equally in the countries. In China, where most of the current suitable habitat for brown bears is found, a loss of 13% of suitable habitat is likely to have a significant impact on the distribution of bears in the country, but much of this change will occur outside the protected areas with a minor change in the habitats inside protected areas. For countries which have small suitable areas for brown bears, loss of suitable habitat may have more profound effects. India for example, which offers a relatively small amount of suitable habitat for brown bears, is likely to experience the greatest impact on brown bear distribution given the significant loss of predicted habitats inside protected areas. A similar situation was observed in Pakistan and Nepal. Therefore, the establishment of future protected areas may be necessary to ensure that the extirpation of bears does not occur in these areas such as India, Pakistan, and Nepal. While suitable habitat is predicted to increase in Uzbekistan and Bhutan, such an increase is unlikely to offset the total loss of habitat to brown bears in Central Asia as those countries have very little suitable brown bear habitat (Aryal et al., 2014). An adaptive approach to establish future protected areas in response to climate‐induced change is necessary to ensure the persistence of the species in this region.

We hypothesize that two bioclimatic variables, annual precipitation and temperature, may significantly challenge the geographical distribution of brown bears in Central Asia by potential direct and indirect effects. These effects are not only anticipated to cause shifts in brown bear distributions, as species often pursue an optimal habitat, but threatening their viability due to range reductions or fragmentations and partially altering their biological systems (Parmesan, 2006). Our results highlighted the influence of future meteorological conditions on behavioral plasticity, the ability to respond to environmental changes, which will dictate how well brown bears can adjust or resist to changes occurring in their environment (Williams, Shoo, Isaac, Hoffmann, & Langham,). For example, brown bears tend to modify their ambulatory activities and movement speed during periods of increased precipitation with an increase of 0.1 km/hr for each increase of 5 mm of rainfall (Martin 2009) which might predict the relatively greater energetic costs of future high‐speed locomotion of brown bears to meet their basic requirements (Gormezano, McWilliams, Iles, & Rockwell, 2016) by the probable increase in precipitation from 406 to 459 mm by 2,050. Similarly, the predicated high precipitation should accelerate the melting of snow, increase the run‐off, and cause streams to overflow. On one hand, this could reduce the period during which snow still offers a protective shelter for optimal denning structure and environment. On the other hand, due to poor winter precipitation, the snow depth and snow cover in alpine scrub and meadow habitats would be very less leading to changes in plant community structure, composition, and biomass in the following spring and summer. This could force individuals to move more in search of better quality habitats increasing energy costs. Furthermore, disturbances resulting in displacement at this stage of the life cycle could have deleterious effects, especially in the presence of altricial bear cubs developing locomotory skills, as a new den site must be found, and the offspring need to be relocated (John, Swenson, Andersen, & Barnes, 2000).

Our results also highlighted the influence of predicted temperature increase from −1.2°C to 1.6°C by 2,050 on brown bear. The global change in temperature will inevitably lead to challenging impacts not only on brown bear distribution patterns but also on their ethological repertoire and cyclic and seasonal changes of biological activities. Generally, mammals can cope with escalating thermal stress by adopting some thermoregulatory behavioral responses (Sawaya, Ramsey, & Ramsey, 2017) including shifting to more nocturnal activities, as a least‐cost thermoregulation strategy, to reduce the costs associated with autonomic temperature regulation (Maloney, Moss, Cartmell, & Mitchell, 2005). Bears are largely diurnal (MacHutchon, 2001) but become less active at daytime and more nocturnal when temperature rises (McLellan & McLellan, 2015). Similarly, failure or inadequacy of the behavioral thermoregulatory measures will inevitably lead to costlier physiological adaptations to climate changes. For instance, increased temperatures have been strongly linked to shorter periods of denning in bears (Inouye, Barr, Armitage, & Inouye, 2000). Shorter durations of hibernations could lead to altered energy budgets, reduced cub survival and fitness and higher incidents of human–bear conflicts (Pigeon, Stenhouse, & Côté, 2016). Finally, an examination of regional studies over a 50‐year period showed that carnivore body sizes have generally increased over the past half‐century. This may be a result from the increases in the length of warm season associated with climate change (Yom‐Tov, 2003). Following this trend, brown bears may also increase in their body size that mandates extra‐energy demands which could threat the predator–prey relationship through magnifying predation effects and reduce the probability of prey coexistence (Thakur, Kunne, Griffin, & Eisenhauer, 2017). An example could also clarify the effect of climate change on predator–prey dynamics in the region (Aryal et al., 2014). Brown bears can prey heavily on small mammals such as pika (*Ochotona* spp.) and marmot (*Marmota* spp.) at high altitudes which are sensitive to temperature and precipitation changes (Francl, Hayhoe, Saunders, & Maurer, 2010); therefore, future climate change may alter their distribution and population dynamics. Pika and marmot, which effectively inhabit high‐elevation “islands,” may have to migrate upwards in elevation in order to live under preferred climate conditions. If climate changes cause reductions in wild prey populations or availability, there may be an increased risk of brown bears switching their feeding strategy to kill more livestock in the region (Aryal et al., 2012, 2010 ), thereby exacerbating human–bear conflict.

Any loss of suitable habitat within protected areas is of concern for brown bear conservation in Central Asia, because it may result in bears moving out of protected area due to climate‐induced range shift (Upward and northward). Such movement may increase encounters with humans and a subsequent increase in human–bear conflicts and increased bear mortalities. However, more research is necessary to determine the impact of climate change on food resources (bottom‐up regulation) and nutrition of bears. As well, the addition of mortality risk to bear models will help to understand top‐down factors that may affect populations (Nielsen, McDermid, Stenhouse, & Boyce, 2010). The data necessary to resolve such nutritional and mortality‐related factors are to our knowledge not available across the entirety of our immense study areas but should be a focus of future research. In addition to upwards migration, future climate change may cause some mammal species to move northward (Francl et al., 2010). The range shift from southern areas such as India, Nepal, and China to northern regions such as Mongolia would be unlikely due to habitat fragmentation and loss (Inkley et al., 2004; Rosenzweig et al., 2008). Such a situation might contribute to local extirpation of brown bear and low genetic diversity (Guralnick, 2006; Hadly et al., 2004). To prevent this, brown bear movement between suitable habitats should be facilitated through the development of corridors which connect habitat between protected areas in different countries (Ramiadantsoa, Ovaskainen, Rybicki, & Hanski, 2015). Such a conservation effort would, of course, be challenging, and require the participation and collaboration of different countries.

## AUTHOR CONTRIBUTIONS

JS, UBS, SCC, SS, MD, ZD, YS, XD, HGF, and LW collected the data. JS and AA analyzed the data, designed the study, and supervised the project. JS, IMH, UBS, SCC, SS, MD, ZD, YS, XD, HGF, LW, and WJ participated in the manuscript writing.

## DATA ACCESSIBILITY

Bioclimatic data are available for download at the databases, www.worldclim.org ; https://www.glcn.org and https://lta.cr.usgs.gov/GMTED2010. Brown bear presence data were uploaded as online (Data S1).
