# Supplementary material for: Decreasing brown bear (Ursus arctos) habitat due to climate change in Central Asia and the Asian Highlands
Source: Ecol Evol. 2018 Nov 20;8(23):11887–99. doi: 10.1002/ece3.4645 (PMC6303720; doi:10.1002/ece3.4645)
Supplement: Supplementary file 1 [file ECE3-8-11887-s001.docx]

| **Table 1. Correlation analysis (Pearson Correlation) of variables to run maxent modelling**  **(Highly correlated variable (r>0.85) were removed to run Maxent modelling).** | | | | | | | | | | | | | | | | | | |  | |  | |  | |  |  | | | |  |  |  |
| --- | --- | --- | --- | --- | --- | --- | --- | --- | --- | --- | --- | --- | --- | --- | --- | --- | --- | --- | --- | --- | --- | --- | --- | --- | --- | --- | --- | --- | --- | --- | --- | --- |
| Correlations | | | | | | | | | | | | | | | | | | | | | | | | | | | | | | | |  |
| Variables | BIO2 | BIO3 | BIO4 | BIO5 | BIO6 | BIO7 | BIO8 | BIO9 | | BIO10 | BIO11 | BIO12 | BIO13 | BIO14 | BIO15 | BIO16 | BIO17 | BIO18 | | BIO19 | | Elevation | | Landcover | | | Slope | | Aspect | | |  |
| Annual Mean Temperature (BIO1) | 0.01 | 0.13 | -0.10 | 0.76 | 0.80 | -0.09 | 0.51 | 0.69 | | 0.69 | 0.80 | 0.32 | 0.28 | 0.02 | -0.15 | 0.28 | 0.01 | 0.17 | | 0.22 | | -0.52 | | -0.43 | | | 0.00 | 0.07 | | | | |
| Mean Diurnal Range (BIO2) | 1.00 | 0.49 | -0.07 | 0.01 | -0.12 | 0.11 | 0.27 | -0.15 | | -0.15 | 0.06 | -0.27 | -0.04 | -0.64 | 0.69 | -0.07 | -0.67 | 0.07 | | -0.52 | | 0.07 | | -0.11 | | | -0.49 | -0.08 | | | | |
| Isothermality (BIO 3) |  | 1.00 | -0.87 | -0.44 | 0.51 | -0.79 | -0.17 | | 0.32 | 0.32 | 0.62 | 0.11 | 0.20 | -0.41 | 0.43 | 0.21 | -0.24 | 0.26 | | -0.17 | | 0.63 | | 0.05 | | | 0.10 | -0.11 | | | | |
| Temperature Seasonality (BIO4) |  |  | 1.00 | 0.55 | -0.64 | 0.98 | 0.45 | | -0.51 | -0.51 | -0.68 | -0.19 | -0.12 | 0.14 | -0.09 | -0.17 | -0.04 | -0.12 | | -0.12 | | -0.77 | | -0.15 | | | -0.36 | 0.07 | | | | |
| Max Temperature of Warmest Month (BIO5 ) |  |  |  | 1.00 | 0.27 | 0.55 | 0.66 | | 0.30 | 0.30 | 0.24 | 0.06 | 0.05 | 0.08 | -0.20 | 0.02 | -0.05 | -0.05 | | 0.14 | | -0.90 | | -0.43 | | | -0.26 | 0.11 | | | | |
| Min Temperature of Coldest Month (BIO6 ) |  |  |  |  | 1.00 | -0.66 | 0.05 | | 0.86 | 0.86 | 0.98 | 0.31 | 0.20 | 0.03 | -0.19 | 0.23 | 0.13 | 0.09 | | 0.35 | | 0.04 | | -0.20 | | | 0.28 | 0.03 | | | | |
| Temperature Annual Range (BIO7 ) |  |  |  |  |  | 1.00 | 0.48 | | -0.51 | -0.51 | -0.66 | -0.23 | -0.13 | 0.04 | 0.01 | -0.18 | -0.15 | -0.12 | | -0.19 | | -0.74 | | -0.17 | | | -0.45 | 0.06 | | | | |
| Mean Temperature of Wettest Quarter (BIO8 ) |  |  |  |  |  |  | 1.00 | | -0.20 | -0.20 | 0.10 | 0.11 | 0.26 | -0.17 | 0.19 | 0.22 | -0.25 | 0.37 | | -0.36 | | -0.71 | | -0.36 | | | -0.29 | 0.03 | | | | |
| Mean Temperature of Driest Quarter (BIO9 ) |  |  |  |  |  |  |  | | 1.00 | 1.00 | 0.82 | 0.25 | 0.08 | 0.12 | -0.29 | 0.11 | 0.21 | -0.17 | | 0.62 | | 0.04 | | -0.12 | | | 0.23 | 0.03 | | | | |
| Mean Temperature of Warmest Quarter (BIO10 ) |  |  |  |  |  |  |  | | 1.00 | 1.00 | 0.82 | 0.25 | 0.08 | 0.12 | -0.29 | 0.11 | 0.21 | -0.17 | | 0.62 | | 0.04 | | -0.12 | | | 0.23 | 0.03 | | | | |
| Mean Temperature of Coldest Quarter (BIO11 ) |  |  |  |  |  |  |  | |  |  | 1.00 | 0.33 | 0.26 | -0.07 | -0.06 | 0.29 | 0.03 | 0.18 | | 0.24 | | 0.08 | | -0.22 | | | 0.21 | 0.01 | | | | |
| Annual Precipitation (BIO12 ) |  |  |  |  |  |  |  | |  |  |  | 1.00 | 0.92 | 0.49 | -0.12 | 0.95 | 0.52 | 0.79 | | 0.42 | | -0.15 | | -0.24 | | | 0.36 | 0.00 | | | | |
| Precipitation of Wettest Month (BIO13) |  |  |  |  |  |  |  | |  |  |  |  | 1.00 | 0.23 | 0.21 | 0.99 | 0.26 | 0.91 | | 0.18 | | -0.16 | | -0.25 | | | 0.20 | -0.01 | | | | |
| Precipitation of Driest Month (BIO14 ) |  |  |  |  |  |  |  | |  |  |  |  |  | 1.00 | -0.69 | 0.26 | 0.93 | 0.10 | | 0.61 | | -0.19 | | -0.06 | | | 0.31 | 0.01 | | | | |
| Precipitation Seasonality (BIO15 ) |  |  |  |  |  |  |  | |  |  |  |  |  |  | 1.00 | 0.16 | -0.66 | 0.28 | | -0.50 | | 0.18 | | -0.01 | | | -0.34 | -0.03 | | | | |
| Precipitation of Wettest Quarter (BIO16 ) |  |  |  |  |  |  |  | |  |  |  |  |  |  |  | 1.00 | 0.29 | 0.91 | | 0.21 | | -0.13 | | -0.25 | | | 0.24 | 0.00 | | | | |
| Precipitation of Driest Quarter (BIO17 ) |  |  |  |  |  |  |  | |  |  |  |  |  |  |  |  | 1.00 | 0.11 | | 0.72 | | -0.04 | | 0.01 | | | 0.41 | -0.03 | | | | |
| Precipitation of Warmest Quarter (BIO18 ) |  |  |  |  |  |  |  | |  |  |  |  |  |  |  |  |  | 1.00 | | -0.18 | | -0.10 | | -0.20 | | | 0.14 | -0.03 | | | | |
| Precipitation of Coldest Quarter (BIO19) |  |  |  |  |  |  |  | |  |  |  |  |  |  |  |  |  |  | | 1.00 | | -0.06 | | -0.07 | | | 0.33 | 0.05 | | | | |
| Elevation |  |  |  |  |  |  |  | |  |  |  |  |  |  |  |  |  |  | |  | | 1.00 | | 0.41 | | | 0.28 | -0.10 | | | | |
| Landcover |  |  |  |  |  |  |  | |  |  |  |  |  |  |  |  |  |  | |  | |  | | 1.00 | | | 0.12 | -0.03 | | | | |
| Slope |  |  |  |  |  |  |  | |  |  |  |  |  |  |  |  |  |  | |  | |  | |  | | | 1.00 | -0.08 | | | | |
